# Supplementary material for: Lifestyle Factors Influencing Metabolic Syndrome after Adjusting for Socioeconomic Status and Female Reproductive Health Indicators: A National Representative Survey in Korean Pre- and Postmenopausal Women
Source: Healthcare (Basel). 2024 Apr 12;12(8):821. doi: 10.3390/healthcare12080821 (PMC11050311; doi:10.3390/healthcare12080821)
Supplement: Supplementary file 1 [file healthcare-12-00821-s001.zip › healthcare-2927699-supplementary.pdf]

Supplementary material

Table S1. Adjusted odds ratio of socioeconomic status, female reproductive health indicators, lifestyle factors, and metabolic syndrome among premenopausal women.

Variables	Categories	OR (95% CI)	*p*
Socioeconomic status			
Age	-	1.018 (0.971-1.067)	.464
Education	Middle school or less	1.262 (0.661-2.410)	.481
	High school	1.145 (0.796-1.646)	.465
	College or higher	1	
Household income	First quintile	2.709 (1.064-4.064)	.032
	Second quintile	1.107 (0.672-1.823)	.064
	Third quintile	1.126 (0.709-1.789)	.690
	Fourth quintile	1.029 (0.919-1.151)	.615
	Fifth quintile	1	
Female reproductive health			
Age of menarche	-	1.029 (0.919-1.151)	.623
Lifestyle facators			
Total calories (kcal/day)	-	1.000 (0.999-1.001)	.498
Total fats (g/day)	-	1.006 (0.984-1.029)	.588
Total proteins (g/day)	-	0.998 (0.975-1.021)	.862
Percentage energy of carbohydrates	-	1.024 (0.995-1.055)	.110
Percentage energy of fats	-	0.991 (0.936-1.050)	.769
Percentage energy of proteins	-	1.020 (0.918-1.134)	.713
Smoking	Current smoker	4.462 (2.307-8.628)	.000
	Ex-smoker	1.207 (0. 629-2.317)	.571
	Non-smoker	1	
High-risk drinking	Yes	1.170 (0.596-2.299)	.648
	No	1	
Regular physical exercise	Yes	1	
	No	1.107 (0.801-1.531)	.538
Obesity	Underweight	0.398 (0.051-3.081)	.378
	Normal	1	
	Overweight	3.965 (2.325-6.763)	.000
	Obese	21.158 (13.511-33.136)	.000

Table S2. Adjusted odds ratio of socioeconomic status, female reproductive health indicators, lifestyle factors, and metabolic syndrome among postmenopausal women.

Variables	Categories	OR (95% CI)	*p*
Socioeconomic status			
Age	-	0.979 (0.929-1.031)	.416
Education	Middle school or less	1.267 (0.781-2.055)	.338
	High school	0.908 (0.584-1.413)	.670
	College or higher	1	
Household income	First quintile	1.391 (0.749-2.583)	.297
	Second quintile	1.155 (0.701-1.905)	.571
	Third quintile	1.263 (0.786-2.030)	.335
	Fourth quintile	1.021 (0.928-1.123)	.852
	Fifth quintile	1	
Female reproductive health			
Age of menarche	-	1.021 (0.928-1.123)	.667
Lifestyle facators			
Total calories (kcal/day)	-	1.000 (0.999-1.001)	.906
Total fats (g/day)	-	0.984 (0.957-1.012)	.251
Total proteins (g/day)	-	1.006 (0.987-1.025)	.556
Percentage energy of carbohydrates	-	0.991 (0.963-1.020)	.536
Percentage energy of fats	-	1.012 (0.951-1.077)	.712
Percentage energy of proteins	-	0.943 (0.853-1.043)	.253
Smoking	Current smoker	1.154 (0.516-2.579)	.728
	Ex-smoker	1.045 (0.456-2.392)	.917
	Non-smoker	1	
High-risk drinking	Yes	0.834(0.344-2.024)	.688
	No	1	
Regular physical exercise	Yes	1	
	No	1.459 (1.049-2.029)	.025
Obesity	Underweight	0.348 (0.046-2.641)	.308
	Normal	1	
	Overweight	3.242 (2.050-5.128)	.000
	Obese	11.282 (7.481-17.013)	.000
